# Supplementary material for: CLASH (Chromatin Loop Across-sample Score Harmonizer) quantifies the relative contributions of genetic variation, methylation, and CTCF occupancy on chromatin loop strength across individuals
Source: bioRxiv. 2026 Jun 4:2026.06.01.729143. Preprint. [Version 1] doi: 10.64898/2026.06.01.729143 (PMC13252114; doi:10.64898/2026.06.01.729143)
Supplement: Supplement 2 [file NIHPP2026.06.01.729143v1-supplement-2.pdf]

bioRxiv preprint doi: https://doi.org/10.64898/2026.06.01.729143; this version posted June 4, 2026. The copyright holder for this preprint (which was not certified by peer review) is the author/funder, who has granted bioRxiv a license to display the preprint in perpetuity. It is made available under a CC-BY 4.0 International license.

Supplemental Methods:

Data processing. Hi-C data were generated for five male lymphoblastoid cell lines (GM19317, GM19347, HG01457, HG02666, and HG03248), each sequenced in three independent runs by Phase Genomics (Seattle, WA), with one additional run being included from previous Human Genome Structural Variation Consortium (HGSVC) data (Logsdon et al. 2025). Raw FASTQ files were aligned to the GRCh38 reference genome and processed using the distiller-sm pipeline, excluding unplaced contigs. PCR duplicates, self-circles, and dangling ends were removed following standard Hi-C quality-control steps.

The lower-limit of contact resolution imposed by molecular byproducts for all samples was 1-2 kb, as summarized using MultiQC (v1.20; (Ewels et al. 2016)). To determine if the sequencing depth of our samples supported analysis at this high resolution, we adapted the Juicer (Durand et al. 2016) script provided by https://github.com/aidenlab/juicer/blob/main/misc/calculate_map_resolution.sh to guide plausible Hi-C resolution analysis for our samples, which yielded map resolutions ranging from

bioRxiv preprint doi: https://doi.org/10.64898/2026.06.01.729143; this version posted June 4, 2026. The copyright holder for this preprint (which was not certified by peer review) is the author/funder, who has granted bioRxiv a license to display the preprint in perpetuity. It is made available under a CC-BY 4.0 International license.

2.45 kb – 2.95 kb (Table S1). These values are reasonable given prior work that has shown that a read depth of ~5 billion read pairs is necessary for Hi-C analysis at 1 kb resolution (Rao et al. 2015), ~1 billion read pairs allows for slightly underpowered Hi-C analysis at 2 kb (Rao et al. 2015; Lee et al. 2022), and ~500 million read pairs is sufficient for 5 kb analysis (Rao et al. 2015). Thus, we generated contact matrices at resolutions as high as 1 kb, but with a focus on 2 kb and 5 kb resolutions for downstream analysis, using the cooler (v0.10.3) framework, and applied iterative correction (ICE) for normalization via cooler balance. Across the samples, cooler balance assigned NaN weights to an average of 12.84% ± 0.26% of bins at 2 kb resolution (1,544,155 bins total) and 13.12% ± 0.30% of bins at 5 kb resolution (617,669 bins total).

Phased variant call files (VCFs) containing both single-nucleotide polymorphisms and structural variants were obtained from the HGSVC Phase 3 dataset, and were generated from the pav2 pipeline (docker://becklab/pav:latest; (Ebert et al. 2021). These VCFs were produced by aligning telomere-to-telomere haplotype assemblies generated from PacBio HiFi and Oxford Nanopore reads to GRCh38, calling variants with dipcall (v0.3) and phasing with WhatsHap (v2.8). The resulting phased VCFs were used to annotate deletions, insertions, and SNPs overlapping CTCF motifs and chromatin loops.

Single-molecule chromatin Fiber-seq data were produced using PacBio Revio HiFi sequencing (30 h movie time per SMRT Cell) at the University of Washington. Each sample achieved a mean genome-wide coverage of 33.5x, an average read length of 20.7 kb, HiFi yields of ~103 Gb, and read-quality scores ranging from Q30–Q33 (Supplementary Table S2). Reads containing pre-annotated N^6-methyladenine (m^6A) modifications were aligned to GRCh38 using pbmm2 (v1.10.0). The resulting BAM files were phased using WhatsHap phase guided by corresponding variant calls. Processed reads were analyzed using the standard fibertools command suite (v0.6.4) pipeline. M^6A bases and nucleosomes were identified using the following command:

```
ft extract GM19317/GM19317_m6_nuc.bam --reference \
  -q -a GM19317_all.tsv -n GM19317_nucleosomes.bed \
  --m6a GM19317/GM19317_m6a.bed -t 8
```

bioRxiv preprint doi: https://doi.org/10.64898/2026.06.01.729143; this version posted June 4, 2026. The copyright holder for this preprint (which was not certified by peer review) is the author/funder, who has granted bioRxiv a license to display the preprint in perpetuity. It is made available under a CC-BY 4.0 International license.

Footprinting was performed against known CTCF motif coordinates (JASPAR MA0139.1) using the following command:

```
ft footprint "GM19317_m6_nuc.bam" \
 --bed "GM19317H1_CTCF_COORDINATES_clean.bed" \
 --yaml "ctcf.yaml" \
 --out "GM19317H1_ctcf_footprint.bed"
```

For each motif, both the total number of fibers spanning the site and the number containing a CTCF-sized footprint were counted. The ratio of footprinted to total fibers defined the CTCF occupancy frequency for that site.

Per-base m^5C CpG methylation calls for all samples were obtained from the HGSVC. Base-called reads were aligned to the telomere-to-telomere assembly of each sample using minimap2 (v2.30-r1287). Methylation profiles were phased using WhatsHap phase (v2.8) guided by dipcall-derived VCFs, yielding haplotype-specific methylation tracks. For downstream integration with Hi-C and Fiber-seq, the number of methylated CpG sites per fiber per genomic bin and per CTCF site was used as a proxy for total methylation signal.

AB compartment analysis. A/B compartments were computed using the Cooltools (v0.7.0) eigs_cis function applied to ICE-balanced Hi-C matrices at 100 kb resolution using the following command:

```
eigvals, eig_df = cooltools.eigs_cis(
    GM19317_100000.cool,
    phasing_track=bins[['chrom', 'start', 'end', 'frac_gc']],
    n_eigs=1
  )
```

GC content was used as the phasing track to orient the first eigenvector (E1), such that positive values correspond to GC-rich, transcriptionally active (A) compartments. The resulting E1 eigenvalues for each genomic bin were compared between samples using pairwise MSE and

bioRxiv preprint doi: https://doi.org/10.64898/2026.06.01.729143; this version posted June 4, 2026. The copyright holder for this preprint (which was not certified by peer review) is the author/funder, who has granted bioRxiv a license to display the preprint in perpetuity. It is made available under a CC-BY 4.0 International license.

sign concordance to quantify compartment similarity. A null expectation for MSE was estimated as the genome-wide variance of E1 values pooled across all samples, corresponding to the expected mean squared difference under random alignment of compartment eigenvectors. For visualization, E1 profiles across a representative 35 Mb region were plotted for all samples. Hi-C maps showing compartment state and inter-sample compartment changes were generated using matplotlib from sample cooler files. For all A/B compartment, TAD domain, chromatin loop, and chromatin interaction-related analyses in this manuscript, we highlight representative loci and regions that illustrate the general trends observed genome-wide.

Chromatin accessibility with Fiber-seq. For all samples, Fiber-seq reads across both haplotypes were partitioned into 100 kb genomic bins (only relevant fragments of each read were kept in the case of reads that overlap multiple bins). For each read or fragment of a read, the total number of adenines and m^6A methylated adenines were determined and summed across every read for every bin. The proportion of methylated adenines/total adenines within each bin was computed. For between-sample comparisons, these percentages were mean-centered within each sample to remove global shifts in modification levels and focus analyses on relative spatial variation along the genome. For each pair of samples, we computed the average MSE between mean-centered m^6A levels at the same genomic bin across samples and sign concordance as the percentage of bins in which both samples showed deviations in the same direction (positive or negative relative to their respective means), excluding bins with 0 deviation. Final statistics reported averaged MSE and sign concordance across each pair of samples. A permutation-based null model was constructed where for each sample pair, m^6A values from one sample were randomly permuted across bins 1,000 times while preserving the empirical value distribution, and the MSE was recomputed to generate a null distribution of similarity values.

Identification of differential interactions using diffHic. Genome-wide differential chromatin interactions between the samples were identified using the diffHic package (v1.38.0) following the protocol detailed in the diffHic User’s Guide (https://bioconductor.posit.co/packages/devel/bioc/vignettes/diffHic/inst/doc/diffHicUsersGuide.pdf). The steps performed were:

bioRxiv preprint doi: https://doi.org/10.64898/2026.06.01.729143; this version posted June 4, 2026. The copyright holder for this preprint (which was not certified by peer review) is the author/funder, who has granted bioRxiv a license to display the preprint in perpetuity. It is made available under a CC-BY 4.0 International license.

1. Generate raw Hi-C contact matrices for the 5 samples at 1 kb, 2 kb, and 5 kb resolution using the cooler dump() function. These matrices were used as input for diffHic.

2. Filter out centromeres, telomeres, interactions < 10 kb, interactions > 1 Mb, and interactions where across all 5 samples, the sum of contacts was < 2.

3. Normalize for bias using the normOffsets() function, which allows for cross-sample pixel comparisons. Following the documentation, the correctedContact() was not implemented as ICE normalization is not necessary for cross-sample pixel comparisons.

4. Estimate dispersion and fitting the quasi-likelihood negative binomial GLM model to the data. This step was repeated with each iteration comparing one sample to the remaining samples, generating one list of differential interactions for each sample. Our setup kept deletions in the Hi-C dataset as 0 counts, allowing them to be identified as differential between samples.

The resulting interaction lists were filtered to retain pixels passing logCPM > –4 in at least one of the five sample and interaction distances between 10 kb and 1 Mb. The logCPM threshold was selected based on the distributions of logCPM and logFC values, optimizing retention of informative contacts while preserving the expected unimodal logFC distribution centered around zero at 5 kb resolution (Supplemental Figures 4-8). Across filtered loci, logFC standard deviations followed a negative binomial distribution with a mean $\sigma = 0.36$, reflecting that most interactions are conserved and a minority are differential. Significant interactions were identified using Bonferroni correction at each resolution, and genome-wide logFC profiles were visualized for each sample. The set of 367 significant interactions at 5 kb was used for mechanistic analyses.

Mechanistic analysis of diffHic differential interactions. Four mechanisms were evaluated for their association with diffHic-identified differential Hi-C pixels (aka interaction-pairs):

1. Large-scale sequence differences between interaction bins
2. Small-scale sequence differences within interaction bins
3. m^6A methylation
4. m^5C methylation

bioRxiv preprint doi: https://doi.org/10.64898/2026.06.01.729143; this version posted June 4, 2026. The copyright holder for this preprint (which was not certified by peer review) is the author/funder, who has granted bioRxiv a license to display the preprint in perpetuity. It is made available under a CC-BY 4.0 International license.

For genetic variation, a leave-one-out (LOO) approach was used to identify the sample with the most extreme value at each differential pixel. For each interaction locus, the sample with a differential number of total bases changed (via SNPs, insertions, and deletions) both between interaction anchor bins and in interaction anchor bins was determined. Samples were considered differential if they exhibited a median absolute deviation (MAD)-based Z-score ≥ 2 and their LOO t-test p-value fell below a Bonferroni-adjusted 0.02 threshold. This was chosen as the threshold because it corresponds to strong outlier deviation in a MAD-based framework while maintaining sensitivity given the sample size (n = 5). For bins with a differential genetic alteration sample we quantified the match rate between that sample and the sample with the largest absolute value logFC. Statistical significance was assessed using a two-sided binomial test under a null probability of 0.2 (reflecting the 1-in-5 chance of a match by random expectation).

M^6A methylation rates were computed by determining, for each fiber and each bin, the number of methylated adenines divided by the total adenines. Rates were averaged across fibers and across both interacting bins for each differential Hi-C contact. To normalize baseline differences between individuals, genome-wide expected methylation ratios were computed and compared to the observed ratios. Bins with observed log-ratios deviating from expectation by > 0.2 were flagged as differential, and the direction of the deviation was recorded. Because increased m^6A methylation is expected to correlate with reduced chromatin contacts, loci were classified into four categories (+m^6A, +logFC; +m^6A, -logFC; -m^6A, +logFC; -m^6A, -logFC) based on whether the differential-methylation sample aligned with the minimum or maximum logFC value. The match rate of each set was calculated and statistical significance was assessed using a two-sided binomial test under a null probability of 0.2. This calculation was repeated while excluding loci where the identified differential sample contained a CTCF binding site within 10 kb of either interacting bin to differentiate the effects of low m^6A values caused by closed chromatin from the effects of low m^6A values caused by CTCF binding.

CpG methylation levels were obtained from phased ONT reads. For each resolution, per-bin methylation fractions were computed as the number of methylated cytosines divided by the total number of cytosines within that bin, separately for each haplotype (H1, H2). The methylation fractions of the two interaction bins were averaged to obtain a single value per

bioRxiv preprint doi: https://doi.org/10.64898/2026.06.01.729143; this version posted June 4, 2026. The copyright holder for this preprint (which was not certified by peer review) is the author/funder, who has granted bioRxiv a license to display the preprint in perpetuity. It is made available under a CC-BY 4.0 International license.

haplotype per differential interaction. To identify whether a sample exhibited aberrant m^{5}C levels at a locus, we applied a similar robust outlier framework as before. Across the 10 haplotypes, methylation values were converted into MAD Z-scores, and the haplotype with the largest absolute Z-score was considered a candidate differential sample. A leave-one-out (LOO) Z-score was then computed by recalculating the median and MAD excluding the candidate haplotype sample. A haplotype was marked as differential if both the MAD Z-score and the LOO Z-score were $\geq$ 2, and the direction of the deviation ("+" for higher methylation, "–" for lower) was recorded. For each differential m^{5}C event, we compared the direction of methylation deviation with the Hi-C log fold-change (logFC) of the haplotype-sample. Because increased CpG methylation is expected to reduce chromatin contacts, loci were classified into four categories (+m^{5}C, +logFC; +m^{5}C, -logFC; -m^{5}C, +logFC; -m^{5}C, -logFC) based on whether the differential-methylation sample aligned with the minimum or maximum logFC value. For each category, we computed the proportion of loci in which the m^{5}C deviation correctly predicted the logFC extremum and assessed significance using a two-sided binomial test with null probability 0.2.

To assess joint explanatory power, differential bins were annotated with any mechanism for which a differential sample was detected, and match-rate analyses (total bases changed between interaction bins and in interaction bins; +m^{6}A, -logFC; +m^{5}C, -logFC) were calculated. To evaluate independence, match-rate analyses were recomputed after removing any differential interactions explained by two or more mechanisms, demonstrating that each mechanism retains significance when controlling for the others. A Venn diagram generated using the venn Python package (v0.1.3) summarized the overlap between the match rates of each mechanism in bins that exhibited at least one differential mechanism.

Next, we computed the frequency with which each unique genomic bin appeared across all differential interactions and quantified the proportion that fell within 10 kb of a chromatin loop in the 2D Hi-C matrix as well as within homozygous bin-eliminating deletions, calculating the null through circular permutation (n = 1000) by randomizing differential interaction anchors at identical genomic distances along the chromosome and testing against fixed homozygous deletion intervals with empirical permutation p-values. We also clustered differential pixels within 10 kb × 10 kb of each other in the 2D Hi-C matrix and plotted the distribution of the

bioRxiv preprint doi: https://doi.org/10.64898/2026.06.01.729143; this version posted June 4, 2026. The copyright holder for this preprint (which was not certified by peer review) is the author/funder, who has granted bioRxiv a license to display the preprint in perpetuity. It is made available under a CC-BY 4.0 International license.

number of differential pixels within each cluster, stratified by clusters within 10 kb of a loop or within homozygous deletions.

CTCF site determination and PWM score calculation. Because 85% of chromatin loops form at CTCF sites (Rao et al. 2015), CTCF motif locations in each sample were derived by running the FIMO software (v5.3.0) on haplotype-resolved assemblies for each sample using the following command:

```
fimo CTCF.meme GM19317.vrk-ps-sseq.asm-hap1.fasta
```

The least stringent CTCF motif instance across all haplotypes retained a FIMO p-value of 3.85×10^{-6}, which approaches the suggested FIMO p-value threshold of $p < 1 \times 10^{-6}$ (Dozmorov et al. 2022) Because this process yielded 55,422 ± 1,180 CTCF sites per haplotype, consistent with prior estimates of expected human CTCF site counts (Chen et al. 2012), we did not further filter CTCF sites to those satisfying $p < 1 \times 10^{-6}$, which would have eliminated ~29,166 sites from each haplotype. We note that although FIMO uses a default p-value threshold parameter (“--thresh”) of $p < 1 \times 10^{-4}$, FIMO also uses a default “--max-stored-scores” parameter of 100,000 sites, and when those 100,000 sites are filled, the weakest sites are truncated to make room for new sites. This led to slight variation in the maximum p-value retained by each haplotype (3.48×10^{-6} – 3.85×10^{-6}) despite running FIMO with identical commands to that shown above. Importantly, we validated that within each haplotype, this truncation process did not remove any CTCF sites that satisfied the maximum p-value after running FIMO with --max-stored-scores set to 1,000,000 sites. Results were mapped back to the reference genome GRCh38 using the Long Read Aligner software (v1.3.7.2). This process successfully mapped ~90% of CTCF sites for each sample back to the reference genome, averaging ~49,983 sites per haplotype.

The FIMO output also included the base sequence for each CTCF site. The position weight matrix (PWM) scores were calculated for each sequence for each haplotype. A PWM probability matrix with per-base probabilities at each position in the motif was constructed from JASPAR’s 2018 MA0139.1 CTCF frequency matrix. For each haplotype-resolved CTCF site, the corresponding 19 bp sequence was scored by summing the position-specific probability weights associated with each base and dividing the total by the motif length to obtain a motif probability

bioRxiv preprint doi: https://doi.org/10.64898/2026.06.01.729143; this version posted June 4, 2026. The copyright holder for this preprint (which was not certified by peer review) is the author/funder, who has granted bioRxiv a license to display the preprint in perpetuity. It is made available under a CC-BY 4.0 International license.

score, which enables direct comparison across the ten haplotypes in our dataset and integrates naturally with downstream quantitative models. Importantly, the minimum PWM scores across all 10 haplotypes were exactly the same (0.514), indicating that the slight variation in maximum p-values between haplotypes did not change which CTCF sequence motifs were considered for downstream analysis.

For each CTCF sequence, the number of bases mutated compared to the canonical 15 bp CTCF consensus sequence 5′-NCANNAGRNGGCRSY-3′ (Hashimoto et al. 2017) was calculated. To accomplish this, the 15 bp window was aligned across each haplotype sequence to identify the highest-scoring local match. Within the best alignment, bases that violated the consensus rules were counted as mismatches, and the number of mismatches per sequence was recorded.

CTCF site m^{5}C methylation. CpG methylation was quantified at single-base resolution using ONT-derived per-nucleotide methylation calls for each haplotype. This data was processed to determine, for each genomic position, the number of reads overlapping that cytosine and the number of reads supporting a methylated call for each sample and haplotype. For each position, a methylation fraction was computed as the number of methylated cytosines / the total number of cytosines. For every haplotype-resolved CTCF motif, all CpG positions falling between the start and end coordinates were retrieved. Multiple CpG methylation events on the same site were averaged, producing a site-level methylation profile for every motif and haplotype.

In parallel, a hidden Markov model (HMM) was applied genome-wide to classify each CpG position as hypomethylated or hypermethylated. These HMM state calls were merged into the CTCF annotations using the same coordinate-based procedure, yielding for each haplotype-specific CTCF site a categorical label of hypomethylated, hypermethylated, or mixed (if CpG positions within the motif belonged to different HMM states). Similar results could be achieved from the Fiber-seq m^{5}C reads, however the ONT reads were used as orthogonal support.

CTCF site accessibility. The m^{6}A rates (the number of m^{6}A adenines / the total number of adenines) were calculated within each 1 kb bin for each sample. The average m^{6}A rate of the four bins (two upstream, two downstream) surrounding each bin that contained a CTCF site was

bioRxiv preprint doi: https://doi.org/10.64898/2026.06.01.729143; this version posted June 4, 2026. The copyright holder for this preprint (which was not certified by peer review) is the author/funder, who has granted bioRxiv a license to display the preprint in perpetuity. It is made available under a CC-BY 4.0 International license.

calculated. To account for technical biases from sequencing, the m^{6}A rates were Z-scored within sample and sigmoid-transformed into a score between zero and one, representing how accessible the chromatin near each CTCF site was compared to the other CTCF sites in the sample. Z-scores were calculated within CTCF sites rather than genome-wide as downstream analysis was focused only on CTCF sites.

Quantifying correlations between genetic and epigenetic factors and occupancy. Each CTCF site across all ten haplotypes was annotated with by PWM score, the number of sequence mutations relative to the consensus motif, the mean CpG methylation level, the CpG methylation state (hypomethylated, mixed, hypermethylated), the average accessibility rate, and the Fiber-seq–derived CTCF occupancy value. To enable cross-haplotype comparisons of the same CTCF site, we matched CTCF sites that mapped back to the GRCh38 reference genome within 50 bp together. Global pairwise correlations between each feature and occupancy were computed to assess individual relationships. To quantify the joint association between sequence strength, accessibility, and CpG methylation and their relationship to CTCF occupancy, we fit an ordinary least squares (OLS) regression model using PWM score and methylation as predictors of occupancy and calculated the correlation between observed and fitted values. PWM scores were min–max normalized to the range [0,1], whereas methylation values were transformed to a z-score across all motif instances. Rows containing missing values for any of the predictors or occupancy were excluded. Because the goal of this analysis was association rather than prediction, the model was fit to the full dataset without a train–test split. Global significance of the association model was assessed using the F-test for linear regression with two predictors. All analyses were performed in Python using pandas, numpy, scikit-learn, scipy, matplotlib, and seaborn.

Loop discovery using Mustache and HiCExplorer. The following commands were used to discover loops, with appropriate length parameters and adjustments per sample:

```
hicDetectLoops -m GM19317_5000.cool -o \
GM19317_HICEX_5000_01.bedpe --maxLoopDistance 2000000 \
 --windowSize 10 --peakWidth 6 --pValuePreselection 0.1 \
 --pValue 0.1
```

bioRxiv preprint doi: https://doi.org/10.64898/2026.06.01.729143; this version posted June 4, 2026. The copyright holder for this preprint (which was not certified by peer review) is the author/funder, who has granted bioRxiv a license to display the preprint in perpetuity. It is made available under a CC-BY 4.0 International license.

```
mustache -f GM19317.mcool -r 2kb -pt 0.1 \
 -o GM19317_2000_01.tsv -p 50 -st 0.7
```

Chromatin loops were initially called on each resolution of each sample using both Mustache (v1.3.3; MUST) and HiCExplorer (v3.7.5; HICEX) software with p-values of both 0.1 and 0.01. Mustache loops called at 1 kb and 2 kb resolutions included an additional st parameter which was set at 0.7 following recommended settings. All HiCExplorer loops called with the hicDetectLoops() function included additional parameters of maxLoopDistance = 2000000, windowSize = 10, and peakWidth = 6. To compare loop sets, we computed Jaccard indices between callers and between samples after expanding each loop to a ±10 kb neighborhood to account for minor positional differences in peak localization. The same comparisons were also performed on two independent GM19317 technical replicates at 10 kb resolution.

We also calculated Jaccard index values with respect to varying sequencing depth by splitting the read pairs of unbalanced GM19317 cooler files at 1 kb, 2 kb, 5 kb, and 10 kb resolutions in half, balancing both output halves, and calling loops on both halves for all four resolutions using Mustache (HiCExplorer failed to call loops on either of the halves at any of the resolutions). Jaccard index values were then calculated between halves at each resolution, as they were above.

CLASH. CLASH is a method that assigns a loop strength score to a provided Hi-C locus based on the structure of Hi-C signal at that locus, accounting for the roughly radial symmetry of decaying contacts from the loop center (Eagen 2018). The input for CLASH is pooled loop call sets of genomic coordinates where a previous method has called a chromatin loop. For each candidate loop, bins within 10 kb of the diagonal were excluded to avoid including windows with invalid ranges of separation and crossing over the diagonal of the Hi-C matrix. Then, for each called loop, the maximum balanced contact count within a dynamic search window ranging from a 5×5 matrix (±2 bins) for short-range loops (<100 kb) up to an 11×11 matrix (±5 bins) for long-range loops (>200 kb) was selected as the refined center of the candidate loop.

bioRxiv preprint doi: https://doi.org/10.64898/2026.06.01.729143; this version posted June 4, 2026. The copyright holder for this preprint (which was not certified by peer review) is the author/funder, who has granted bioRxiv a license to display the preprint in perpetuity. It is made available under a CC-BY 4.0 International license.

For each refined loop center, we extracted a local Hi-C submatrix with adaptive sizing. Shorter loops (loop anchor separation < 35 kb) always used a 5×5 window (±2 bins). For longer loops, the following procedure was implemented:

1) Start at a minimum radius r = 2, corresponding to the 5x5 ring centered around the loop center.
2) The average balanced contact count of pixels, λ, in the rings corresponding to r, r+1, and r+2 was determined.
3) One sided Welch's t-tests were used to determine if $\lambda_r > \lambda_{r+1}$ or if $\lambda_r > \lambda_{r+2}$ with a p-value ≤ 0.05.
4) R is incrementally increased and the process is repeated from step 2. Matrix expansion stops when three consecutive radii fail the test in step 3, returning r = max(2, the max r that passed the test in step 3).
5) The (2r + 1, 2r + 1) matrix centered around the refined loop center is extracted.

Six features were included to describe each putative loop:

1) Loop anchor separation: The genomic distance (bp) between the two interacting bins.
2) Center value: the balanced contact count of the center pixel for each matrix.
3) Loop prominence: The Z-score of the size of matrix extracted around the loop via our adaptive procedure, computed relative to the matrices extracted for the other samples at the same locus.
4) Zero fraction: the ratio of pixels within the matrix that have a balanced contact count = 0.
5) Smoothness, determined by:
    a) Calculating the Euclidean distance of each pixel in the matrix from the loop center and grouping pixels at the same distance.
    b) Calculating the mean contact intensity of each radial distance to produce a radial intensity profile.
    c) Fitting a univariate spline to the intensity profile.
    d) Calculating the mean squared residual between the observed radial intensities and the fit spline.
6) LC scores: the maximum local contrast between a candidate loop and its background, calculated from Gaussian-smoothed interaction maps across multiple spatial scales. Our

bioRxiv preprint doi: https://doi.org/10.64898/2026.06.01.729143; this version posted June 4, 2026. The copyright holder for this preprint (which was not certified by peer review) is the author/funder, who has granted bioRxiv a license to display the preprint in perpetuity. It is made available under a CC-BY 4.0 International license.

implementation was heavily based on Mustache's implementation (Roayaei Ardakany et al. 2020) and calculated 23 LC scores across between 24 scales at each putative loop, of which we chose the maximum.

An XGBoost binary classifier model, CLASH, was trained using these six features to predict a loop or no loop, minimizing binary cross-entropy. The model used 300 trees with a maximum depth of 4, a learning rate of 0.05, and subsampling of 0.8 for both rows and features. Training data consisted of 1,000 putative loops across 200 loci (199 of which were identified by either/both of Mustache or HiCExplorer in at least one sample, with the other locus, chr7:96076000-97028000, being added after visual observation of the Hi-C map despite not being called in either Mustache or HiCExplorer in any sample). Loops across these 200 loci were manually assigned binary labels of 0 (no loop) and 1 (loop) based on visual inspection of the corresponding Hi-C maps, with 845 true loops spread out across 199 loci and 155 false loops spread out across 71 loci. Our curated set incorporated loci with and without CTCF anchor sites, and with loop anchor separations spanning genomic distances of 36,000-1,214,000 bp. Similar procedures of manual loop presence validation have been used by other machine learning methods for loop detection (Salameh et al. 2020).

Model performance was evaluated using out-of-fold prediction from five-fold cross-validation with grouping by locus to prevent different loops from the same locus appearing in both training and validation sets. Feature importance was determined using SHAP (Lundberg and Lee 2017). For each classification the logit was min-max scaled to yield a continuous CLASH score, and the decision boundary was determined by mapping the classification probability that maximized Youden's J (true positive rate - false positive rate) to min-max scaled space.

Although the primary benefit of CLASH is that it provides this continuous score, we partitioned CLASH scores into five groups to facilitate their visual interpretation on Hi-C maps. Roughly, scores from 0-0.4 represent the absence of a loop, scores from 0.4–0.55 represent non-loop regions with limited enrichment, scores from 0.55-0.65 – which straddle the decision boundary of ~ 0.6 (calculated from the operating point that maximized Youden's J) for this dataset – represent ambiguous loops, scores from 0.65 - 0.85 represent loops, and scores from

bioRxiv preprint doi: https://doi.org/10.64898/2026.06.01.729143; this version posted June 4, 2026. The copyright holder for this preprint (which was not certified by peer review) is the author/funder, who has granted bioRxiv a license to display the preprint in perpetuity. It is made available under a CC-BY 4.0 International license.

0.85 - 1 represent strong loops; these ranges are intended as visual guides rather than strict thresholds.

CLASH Validation:

We validated CLASH's testing AUROC by permuting labels and retraining the model, and we compared CLASH's AUROC classification performance to XGBoost and Logistic Regression models that exclude LC scores as a feature, and only include LC scores as feature, as well as the classification performance of raw LC scores. To enable fair comparisons, we tested the recall for CLASH and the Logistic Regression LC at a matched false positive rate. We also computed the recall and false positive rate of both Mustache and HiCExplorer on the loci comprising the training set.

To demonstrate CLASH's ability to harmonize across samples using the decision boundary corresponding to the maximum Youden's J from the full dataset, we evaluated how many training loci initially called by Mustache and HiCExplorer were worsened, improved, or perfectly improved by CLASH loop predictions (evaluated out-of-fold) and compared this to the LC model performance.

To validate the usage of CLASH scores, we computed the error severity of out-of-fold misclassifications on the training set, defining error severity as the normalized distance from the decision boundary relative to the extent of the incorrect region, and compared the results to those of LC model scores. We also performed orthogonal tests to externally validate CLASH loop-scoring performance by computing Pearson's correlation efficients between CLASH scores with CTCF occupancy globally and within-locus across samples, and compared this to the LC model score correlations.

Quantifying the correlation between CTCF occupancy and CLASH loop-score. To quantify how CTCF protein occupancy globally relates to chromatin loop strength, CLASH-scored loops were first filtered to retain only those in which both loop-associated interaction bins contained a CTCF site within 10 kb. For each loop, the CTCF occupancy values of its two CTCF sites were averaged to generate a single occupancy value. If CTCF occupancy was missing, the CTCF

bioRxiv preprint doi: https://doi.org/10.64898/2026.06.01.729143; this version posted June 4, 2026. The copyright holder for this preprint (which was not certified by peer review) is the author/funder, who has granted bioRxiv a license to display the preprint in perpetuity. It is made available under a CC-BY 4.0 International license.

occupancy value from the other site was used, and if both were missing then the datapoint was excluded from analysis. This per-loop occupancy metric was then paired with the corresponding CLASH loop score, and Pearson's correlation coefficients were computed across all loci and samples to assess the global relationship between CTCF binding and loop strength.

We also quantified the distribution of Pearson's correlation coefficients between CTCF occupancy and CLASH loop-strength between samples at each locus. Loci with fewer than four samples containing both valid occupancy and loop score measurements were excluded. We collected all per-locus correlations and performed a binomial sign test to calculate statistical significance. All analyses were conducted in Python using Pandas, Numpy, Scipy, Seaborn, and Matplotlib.

Quantifying the correlation between PWM scores, m^{5}C methylation, CTCF occupancy, and CLASH loop scores. For each chromatin loop in each sample, PWM scores and CpG methylation levels from the two loop-associated CTCF sites were averaged to generate per-loop measures of motif strength and methylation. If multiple CTCF sites were located within 10 kb of a loop anchor, we chose CTCF sites by sequentially optimizing for CTCF orientation (such that anchors would have convergent orientations (Rao et al. 2015), motif strength (PWM scores), and minimal distance from the anchor bin. This process resulted in 79% of our CTCF-associated loop set being anchored by CTCF sites in convergent orientations. Orientation relationships between paired loop-anchor CTCF sites were highly conserved across haplotypes for the same loop locus, with only 0.2% of loops exhibiting variation in orientation state across haplotypes. These values were paired with CLASH loop scores and occupancy values from earlier, and global Pearson's correlation coefficients were computed to quantify the individual relationships between motif strength and loop intensity. Due to the skewed distribution of methylation values, we stratified loop score distributions by methylation values and used Mann Whitney U tests to quantify the individual relationships between methylation and loop intensity.

We next quantified the number of loci where the CLASH score varied by 0.3, indicating a substantial difference in loop strength, and where occupancy, PWM scores, and methylation data existed for at least 3 of the 5 samples. From this set, we calculated the proportion of loci where the observed difference in loop strength could be explained (defined as having a correlation of $\geq$ 0.25) by each of the three mechanisms.

bioRxiv preprint doi: https://doi.org/10.64898/2026.06.01.729143; this version posted June 4, 2026. The copyright holder for this preprint (which was not certified by peer review) is the author/funder, who has granted bioRxiv a license to display the preprint in perpetuity. It is made available under a CC-BY 4.0 International license.

To test whether PWM scores and methylation values provided explanatory power beyond CTCF occupancy, we compared the correlation between occupancy and loop-strength to the multiple correlation of a combined association model incorporating occupancy, PWM scores, and CpG methylation using Steiger's test for dependent correlations, which accounts for shared outcomes and predictor non-independence. This analysis provided a direct statistical assessment of whether adding genetic (PWM) or epigenetic (m^5C) features improved explanatory power relative to occupancy alone. We performed an analogous comparison using PWM scores and methylation values as the only predictors to evaluate their joint contribution independent of occupancy.

Mediation analysis. To quantify how much of the effect of CTCF motif strength (PWM score) and CpG methylation on loop strength is transmitted through CTCF occupancy, we performed a global product-of-coefficients mediation analysis. For PWM scores, we only pooled observations from variant CTCF loci. We treated PWM scores or CpG methylation as the predictor, CTCF occupancy as the mediator, and CLASH loop strength as the outcome. For each predictor, mediated effects were estimated using linear regression to obtain the product of the predictor-occupancy (a) and occupancy-loop (b) paths, along with the corresponding direct (c') and total (a×b+c') effects and the proportion of the total effect mediated. The percent mediated was calculated as (a×b)/(a×b+c') and averaged across all 5 samples. Uncertainty was measured using a pooled nonparametric bootstrap in which loci were resampled within each sample and per-sample indirect effects were recomputed, averaged across samples for each bootstrap iteration (1,000 iterations), and compared to the mean total effect to derive bootstrap confidence intervals.

Structural variation. We investigated whether large SVs (insertions and deletions) that introduce or remove CTCF binding sites lead to measurable changes in chromatin loop formation. Structural variant calls for each sample haplotype, aligned to the GRCh38 reference genome, were filtered to retain only those altering at least 19 bp (the length of the CTCF consensus motif). FIMO motif scanning was then performed on each SV sequence to identify the number and positions of CTCF motifs gained or lost. We only considered CTCF sites that passed a FIMO p-value threshold of 3.85e-6, matching the least stringent p-value threshold used to identify non-variant CTCF sites across the haplotypes. These SV-associated motif changes were

bioRxiv preprint doi: https://doi.org/10.64898/2026.06.01.729143; this version posted June 4, 2026. The copyright holder for this preprint (which was not certified by peer review) is the author/funder, who has granted bioRxiv a license to display the preprint in perpetuity. It is made available under a CC-BY 4.0 International license.

subsequently intersected with CLASH-derived loop scores to assess whether the addition or removal of CTCF sites corresponded to loop strengthening or weakening.

For each loop locus, and for each sample, we then determined whether either haplotype carried an SV that added at least one new CTCF site (insertion) or removed at least one CTCF site (deletion) within either loop-associated interaction bin. Across the individuals, this yielded two groups of loop strength measurements at each locus: (i) samples whose haplotypes contained a CTCF-altering SV at the interaction bin, and (ii) samples without such an SV. For each locus and for each SV class, we computed the change in loop strength as the difference between the mean CLASH loop score of the SV group and the non-SV group. Statistical significance of these differences genome-wide was assessed using a Wilcoxon test comparing loop scores between SV and non-SV samples at each locus. We later relaxed the FIMO p-value threshold to 1e-5 as part of an exploratory search to include additional loop-associated CTCF variants.

Alpha Genome. To evaluate whether sequence variation alone is sufficient for current sequence-to-map models to accurately predict observed loops and assess whether they could benefit from incorporating methylation and CTCF occupancy data in addition to sequence data, we used Alpha Genome (commit 6973cfe32c8f7d692350b2063c1f7cd611d1cd4f, (Avsec et al. 2026) to predict contact maps for all our haplotypes based on their genetic sequence. We focused on generating maps around the three loci that served as examples for instances where CTCF occupancy (Supplementary Figure 32), SNPs in CTCF sites (Supplementary Figure 34), and methylation in CTCF sites (Supplementary Figure 35) affected loop formation.

To generate the maps, we first extracted the 1,048,576 bp of GRCh38 centered around the midpoint of each pair of interacting bins that formed each loop that we were focused on. We then adjusted the input sequence using variant calls for each of our haplotypes, re-centered the locus of interest, and re-adjusted the overall length of the sequence to be 1,048,576 bp. The adjusted sequence of each haplotype was then used to predict the corresponding contact map using the following function call:

```
output = dna_model.predict_sequence(sequence=hap_seq,
requested_outputs=[dna_client.OutputType.CONTACT_MAPS],
ontology_terms=["EFO:0002784"])
```

bioRxiv preprint doi: https://doi.org/10.64898/2026.06.01.729143; this version posted June 4, 2026. The copyright holder for this preprint (which was not certified by peer review) is the author/funder, who has granted bioRxiv a license to display the preprint in perpetuity. It is made available under a CC-BY 4.0 International license.

The output was then plotted as a Hi-C map in a manner similar to the original examples, including cropping the dimensions of the output to visualize the same region as the original examples. We were unable to extract a matrix-like quantity from the output in order to use CLASH to score the predicted loops.
